# The Measurement-Unit Bias: People Walk or Drive Less to Save a Constant Money Amount When Answering in Meters Compared to Miles

**DOI:** 10.3390/bs15030369

**Published:** 2025-03-14

**Authors:** Nir Reich, Ofer H. Azar

**Affiliations:** Department of Business Administration, Ben-Gurion University of the Negev, Beer Sheva 84105, Israel; azar@bgu.ac.il

**Keywords:** measurement units, time valuation, consumer behavior, traveling cost, heuristics and biases, judgment and decision making

## Abstract

Traditional economic theory suggests that when consumers decide whether to exert effort and travel to a remote store that is cheaper, the decision should compare the time and effort of travelling the relevant distance to the money that can be saved. Our research examined whether the unit of distance measurement, meters or miles, affects the actual distance an individual is willing to travel to save a certain amount of money. We studied the cases of both walking and driving to the remote store. We found in both cases that participants were willing to travel a greater distance for the same amount saved when they answered in miles. This supports our hypothesis, grounded in the literature on heuristics and biases, that the nominal value (which is smaller in miles) affects decisions even though it should be irrelevant from a rational perspective. We denote this behavior as the Measurement-Unit Bias. These findings have important implications for consumer behavior and marketing strategies.

## 1. Introduction

According to classical economic assumptions, when people make decisions, they are expected to base their choices on maximizing their utility function subject to a budget constraint, which means they should weigh costs against benefits. However, over the last few decades, many instances have been documented where people exhibit biased decision-making that deviates from that prescribed by traditional economic models and instead employ various heuristics and biases in their decision-making (see for example Kahneman and Tversky (1979), Tversky and Kahneman (1981), Kahneman (2003), Shah and Oppenheimer (2008) and DellaVigna (2009)).

One of the important common decisions people face is how to trade off effort and money—specifically, how much effort to invest in order to save money. This trade-off is relevant in various contexts, such as when deciding about how many hours to work, how much effort to invest in searching for the lowest price among multiple sellers, whether to travel to a distant but cheaper store, and more. Understanding how people balance effort and money is valuable because it can help individuals improve their decision-making and assist firms, policymakers, and others in responding optimally to the decision-making processes of consumers and citizens.

We contribute to the literature on the trade-off between effort and money by analyzing the influence of the measurement units (meters or miles) on the distance one is willing to cover to save a predefined amount of money. According to classical economic theory, the trade-off between effort (e.g., the distance one is willing to travel) and money (e.g., the amount saved by going to a cheaper store) should not be affected by the unit of distance (meters or miles). Our study aimed to explore whether this principle holds in real decision-making or whether a bias exists in these decisions. Because decisions related to the trade-off between effort and money are common, it is important to establish whether people exhibit a bias and are affected by factors that should be irrelevant like the measurement units of distance. Furthermore, as many trade-off decisions require individuals to process information in specific measurement units, understanding whether these units influence decision-making becomes even more critical. This relevance extends beyond trade-off scenarios, as individuals frequently encounter situations in everyday life where they must interpret and act upon information presented in specific measurement units.

We are unaware of any previous article that studies whether decision-making about the trade-off between effort and money (or economic decision-making more generally) is affected by the measurement units of distance. The article most similar to our study is one on money illusions, which shows a different example of an effect of numerical values that should be irrelevant. The literature on money illusion distinguishes between nominal and real monetary values, arguing that although people should base their decisions on real values, their choices are often influenced by nominal values. For example, Raghubir and Srivastava (2002) and Saayman et al. (2022) showed that tendencies for overspending or underspending depend on the face value of foreign currencies. Wertenbroch et al. (2007) demonstrated that consumers perceive the price premium of expensive name brands as smaller when prices are presented in a currency with lower numerosity (euros) compared to a currency with higher numerosity (pesetas). Majumder et al. (2024) have shown that people are more influenced by the money illusion than they realize. They are less likely to purchase when nominal prices are high, even when the real value remains the same. Darriet et al. (2020) found that financial literacy reduces the susceptibility to money illusion, whereas numeracy alone does not.

Kooreman et al. (2004) found that in the Netherlands, charitable donations increased in average value and showed greater diversity in coin donations following the introduction of the euro. Bittschi and Duppel (2015) found the same phenomenon in Germany, observing changes in donations following the transition to the euro. Brunnermeier and Julliard (2008) studied the link between inflation and housing prices and showed that the housing price–rent ratio is affected by the nominal interest rate instead of the real one.

Even when individuals understand the real value of money and can identify the economically optimal alternative, their judgments may still be influenced by nominal values. Shafir et al. (1997) demonstrated that although respondents could correctly identify the superior economic choice, their reported happiness and likelihood of switching jobs were influenced by nominal values. Ziano et al. (2021) repeated the same experiment and obtained similar results. de Moraes Ferreira et al. (2024) found that the same findings also held true for Brazilians. Shimizu (2019) showed that although high numeracy individuals were able to distinguish between nominal and real values, their decisions regarding well-being were influenced by nominal values. Bawuah (2025) demonstrated how currency redenomination, by altering the nominal value of the currency, can lead to changes in consumption patterns.

Additional studies found that units of measurement or scales can affect decisions. Larrick and Soll (2008) show that people make different decisions when they are given information about miles per gallon fuel consumption versus gallons per 100 miles fuel consumption. Frederick and Mochon (2012) suggest that anchoring is often best interpreted as a scaling effect: the anchor changes how the response scale is used and not how the focal stimulus is perceived.

Our study used scenarios where decision-makers can save money by traveling to a distant store. The participants are asked to decide how much distance they are willing to travel (walking in one scenario and driving in the other) in order to save a predefined amount of money, with the distance being measured in meters or miles (in a between-subjects design). This allowed us to explore, for the first time, whether people behave differently when considering effort in different measurement units. We hypothesized that when participants have to choose the distance they are willing to cover to reach the distant store and save money, they will be willing to cover a shorter distance when evaluating the distance in meters than when evaluating it in miles. This hypothesis is based on the literature that suggests that people are influenced by nominal values. Because responses in meters involve larger numbers compared to responses in miles, we expect the same distance expressed in meters to be perceived as longer than the corresponding distance in miles.

Given that decisions involving the trade-off between effort and money are common in daily life, our research addresses an important gap and offers original and valuable insights.

## 2. Working Hypothesis

The finding in the money illusion literature that people’s judgments are influenced by nominal monetary values led us to hypothesize that a similar phenomenon may exist in decisions involving numbers that are not monetary values. In particular, if we change the measurement unit from meters to miles, different numbers express the same actual distance. We can then test whether the actual distance is the only variable that affects decisions, as it should be, or whether people exhibit a bias and respond to the numbers beyond the actual distance. In particular, we study this using the trade-off between effort and money when the effort is described with different measurement units, either meters or miles. The choice of miles (rather than kilometers) was made because the participants were British and long distances in the UK are expressed in miles. We consulted a guide on units of measure used in the UK on https://www.ukentry.com/units-of-measure-used-in-the-uk.html (accessed on 5 March 2025) and accordingly chose meters as the short unit of distance. We considered the scenario where the buyers identify a specific item they want to purchase but must decide whether to buy it at the current store they visit or at a cheaper, more distant store that requires additional effort to get to it.

We hypothesized that when subjects are asked to determine the distance in meters they are willing to cover to reach the distant store and save money, they will value their time differently and will be willing to cover a shorter distance than when evaluating the distance in miles. The reasoning behind this hypothesis is that people tend to be influenced by nominal values, and since answering in meters results in larger numbers compared to miles, the same distance expressed in meters will be perceived as longer than the equivalent distance in miles. Based on the literature on nominal value biases, we hypothesize as follows:

**Hypothesis:**  *To save a certain amount of money by going to a cheaper store, people are willing to travel a shorter distance when it is expressed in meters than when it is expressed in miles.*

## 3. Experiment: Travel Distance for a Predefined Saving in a Cheaper Store

### 3.1. Materials and Methods

To examine the influence of nominal values through the use of different measurement units on the valuation of effort, we used an online recruitment platform (Prolific) and recruited 100 British participants, who were paid for their participation. We distributed a questionnaire (using Qualtrics) that included two buying scenarios. The experiment was conducted using a between-subjects design, with participants being randomly assigned to one of two treatments, which differed only by the measurement units used in the buying scenarios. Participants were asked to estimate the distance they were willing to travel in order to save 10 pounds on a purchased good. One group received the “meters treatment” and answered in meters, whereas the other group received the “miles treatment” and answered in miles. Each treatment included two scenarios to test the robustness of our findings: in one scenario, participants were asked how far they were willing to walk, and in the other, how far they were willing to drive. The order of these two questions was randomized between participants. Appendix A contains the results of OLS regressions in which we found no significant effect of the question order on the results.

In particular, subjects were asked the following questions (the second treatment in brackets):

*1. Assume that you want to buy a certain product and you found it in a store. However, you can save £10 by walking to a cheaper store that sells the exact same product. How many meters [miles] are you willing to walk to save these £10? ___________meters [miles]*

*2. Assume that you want to buy a certain product and you found it in a store. However, you can save £10 by driving to a cheaper store that sells the exact same product. How many meters [miles] are you willing to drive to save these £10? ___________ meters [miles]*

### 3.2. Results and Discussion

Each of the 100 participants answered the two buying scenarios above, both scenarios in the same treatment (either meters or miles). In one case, we received an extreme, completely unreasonable response (being willing to walk and drive 1947 miles to save 10 pounds) that we had to omit, so we ended up with 99 observations, comprising 46 men and 53 women. The participants’ ages ranged from 25 to 76, with a median age of 43 and a mean age of 43.9. A significant majority of the participants (88 individuals) had not taken any academic courses in economics, and the maximum number of economics courses taken by any participant was five. We converted the distances reported by participants in the miles treatment to meters to allow us to analyze the results. The conversion was achieved by multiplying their miles response by 1609.34. For example, participants who were willing to walk three miles were considered willing to walk 4828.02 m (3 × 1609.34).

To analyze the effect of the measurement unit, we conducted a t-test for the difference in means for each scenario (walking and driving), comparing the distance participants were willing to cover between the two treatments (meters versus miles). The results of the t-test for the walking scenario showed that the average distance participants were willing to walk to save 10 pounds differed based on their response unit. For the group that answered in miles (*n* = 50) the average distance was 3218.68 m (after conversion), and for the group that answered in meters (*n* = 49) the average distance was 1303.04 m. The difference between the two groups was 1915.64 m and was statistically significant, *t*(97) = 6.25, *p* = 0.0000 (two-tailed), indicating that participants who responded in miles were willing to walk a significantly greater distance.

Similarly, for the driving scenario, the average distance participants were willing to drive to save 10 pounds also differed based on their response unit. For the group that answered in miles (*n* = 50), the average distance was 11,201.01 m (after conversion), and for the group that answered in meters (*n* = 49), the average distance was 5041.06 m. The difference between the two groups was 6159.95 m and was statistically significant, *t*(97) = 3.87, *p* = 0.0002 (two-tailed), indicating that participants who responded in miles were willing to drive a significantly greater distance.

These results suggest that there is a statistically significant effect of the measurement units. Participants who answered in miles were willing to cover substantially longer distances than those who answered in meters in both the walking and driving scenarios. Our hypothesis that people are influenced by the measurement unit is therefore supported by the data. In other words, people are willing to exert less effort to save 10 pounds when responding in meters. The reason is that when answering in meters the responses involve larger numbers, and people are affected by these numbers and therefore perceive driving or walking 1609.34 m as more effort than driving or walking one mile, even though the two are in fact the same.

As a robustness check, and to mitigate the effect of extreme observations, we conducted an additional nonparametric test—the Mann–Whitney test. Table 1 and Table 2 present the results of this analysis.

The Mann–Whitney test yielded the same conclusion regarding the effect of the measurement units. Participants who responded in miles were willing to cover longer distances compared to those who responded in meters. This consistency across tests further corroborates the robustness of our findings. We denote this behavior as the Measurement-Unit Bias.

To further analyze the effect of the nominal value, we ran regressions with the dependent variable being the distance the participant was willing to cover (for each scenario, walking and driving), and the explanatory dummy variable being Meter-version. The regression results, reported in Table 3, show the substantial and statistically significant effect of the measurement units.

Additionally, to assess the impact of our results, we performed a power analysis and calculated effect sizes. For the walking scenario, Cohen’s *d* was 1.255, and for the driving scenario, Cohen’s *d* was 0.777. These analyses indicate that the effect is indeed significant, and they further support the strength and reliability of the results. Regarding the power analysis, for an alpha of 0.05 and power of 0.8, with an effect size of *d* = 0.8, the required sample size per group for two-sample *t*-test is 26. With our actual observed differences in means and standard deviations, the required sample size per group was 12 for the walking scenario and 27 for the driving scenario.

To investigate whether gender and age have a significant effect on the outcomes, we ran additional regressions controlling for age, gender and the interaction between age and meter-version and between gender and meter-version. The results, presented in Table 4, show that age and gender do not have a statistically significant effect on the outcomes. This implies that the distance the subjects are willing to walk or drive is not affected by age or gender. In addition, the interaction of age and gender with meter-version is not statistically significant, suggesting that the effect of the measurement unit on the willingness to walk or drive does not differ by age or gender.

## 4. Conclusions

We studied the trade-off between money and effort (as reflected in the distance one is willing to travel to save) in the context of deciding whether to travel to a distant store to save money. We focused on how the measurement units—meters versus miles—affect people’s willingness to exert effort. We built on prior literature about the money illusion, which suggests that nominal values influence decisions. Accordingly, we hypothesized that when distances are expressed in meters and therefore involve higher numbers compared to miles, people would perceive the effort as greater and be willing to travel shorter distances to save a given amount of money (compared to distances expressed in miles).

We conducted an experiment with British participants who were divided into two groups, one responding in meters and the other in miles. The findings support our hypothesis: participants who responded in meters were willing to travel shorter distances for the same monetary savings, a behavior that we denote the Measurement-Unit Bias. We obtained this result with two scenarios, one involving walking and the other involving driving. This confirms that nominal values can influence people’s decisions about effort and money.

The theoretical implications of our research include a deeper understanding of how measurement units and consequently nominal values affect the trade-off between effort and money. This is significant because people make decisions involving this trade-off on a daily basis. For example, individuals decide how many hours to work, or which stores to shop at—closer but more expensive stores versus distant but cheaper ones. The decision of how much effort to invest in searching for better prices at additional stores is also an example of this trade-off. Our research enriches the existing literature by highlighting the effects of the measurement units on such decisions.

Moreover, in economic models of consumer search, consumers have to search for prices by making the effort of travelling to stores to find their prices (the cost of this effort is denoted “search costs”). If consumers make decisions about this effort in a biased manner due to the Measurement-Unit Bias, this can have implications for search models. Similarly, in models of location differentiation of firms, consumers have to incur the costly effort of travelling to the firms in order to buy (in the terminology of these models, the consumers incur “transportation costs”) (Tirole, 1988). Once again, if consumers’ decisions about this effort of traveling to the firm are affected by the Measurement-Unit Bias, this has implications for these location differentiation models. As an example of how consumer biases can complement search and location differentiation models to produce important insights, consider the price dispersion puzzle (Azar, 2013). Theoretically, search and location differentiation models suggest that price dispersion is a function of search and transportation costs, but is independent of the good’s price. Empirical evidence, however, suggests otherwise: price dispersion and price are strongly correlated. This discrepancy has been denoted “the price dispersion puzzle”. Incorporating in the theoretical models the bias of relative thinking (people behave as if their search or transportation costs are increasing the good’s price) can solve the puzzle. By shedding light on the interplay between measurement units and decision-making, our research not only extends the knowledge about how people make effort–money trade-offs, but also paves the way for enhancing economic models to more accurately reflect real-world behavioral patterns.

This research also has practical implications. For example, it suggests that consumers should be educated about the bias we identified so they can make more informed decisions. Greenberg and Shtudiner (2016) and Hsu et al. (2021), for example, demonstrated how education can reduce biases. Firms, on the other hand, may also use the findings to their benefit. For example, a store that is remote from shoppers and cheaper, when making advertisements that mention its distance, should cite the distance in miles or kilometers and not in meters. Similarly, a store that saves the shoppers’ time by being closer to them should advertise the distance it saves travelling in meters and not miles or kilometers. We expect similar findings to ours with other measurement units, and so our findings also have implications for how firms should price products (e.g., per 100 g or per kilogram) or how they should express time (in minutes or hours).

In terms of ideas for future research, one idea is to explore the influence of nominal values with other units, such as weight (grams versus kilograms) or time (minutes versus hours). In addition, it would be interesting to examine how the purpose of the purchase influences the trade-off between effort and money; for example, whether the effect of nominal values is stronger or weaker when buying for someone else, or when purchasing in a professional context. Lastly, a potential study could investigate whether the influence of measurement units persists in situations where there is an objectively correct answer. The goal would be to examine whether certain units lead participants to make decisions that are closer to the correct answer.

## Figures and Tables

**Table 1:** Mann–Whitney test walking scenario.

	Observations	Rank Sum	Expected
Miles	50	3395	2500
Meters	49	1555	2450
Combined	99	4950	4950

*z* = 6.308; *Prob* > $\left|z\right|=0.0000$.

**Table 2:** Mann–Whitney test driving scenario.

	Observations	Rank Sum	Expected
Miles	50	3235.5	2500
Meters	49	1714.5	2450
Combined	99	4950	4950

*z* = 5.164; *Prob* > $\left|z\right|=0.0000.$

**Table 3:** OLS regression.

	(1—Walking Scenario)	(2—Driving Scenario)
Dependent variable	Distance Walking	Distance Driving
Independent variables		
Meter-version	−1915.64 *** (*p* = 0.000)	−6159.95 *** (*p* = 0.000)
Constant	3218.68 *** (*p* = 0.000)	11,201.01 *** (*p* = 0.000)
N	99	99
$R^{2}$	0.287	0.134
*F*	39.01	14.94
*Prob* > *F*	0.000	0.000

Comments: *p*-values are reported in parentheses. *** designates statistically significant at the 0.001 level. Meter-version is a dummy variable that equals 0 in the miles treatment and 1 in the meters treatment.

**Table 4:** OLS regressions controlling for gender and age.

	(1—Walking Scenario)	(2—Driving Scenario)	(1—Walking Scenario)	(2—Driving Scenario)
Dependent variable	Distance Walking	Distance Driving	Distance Walking	Distance Driving
Independent variables				
Meter-version	−1877.35 *** (*p* = 0.000)	−6085.41 *** (*p* = 0.000)	−2374.70 * (*p* = 0.045)	−7903.54 (*p* = 0.203)
Female	−492.00 (*p* = 0.113)	−798.72 (*p* = 0.622)	−311.06 (*p* = 0.476)	167.18 (*p* = 0.942)
Female × Meter-version			−326.68 (*p* = 0.601)	−1796.81 (*p* = 0.585)
Age	2.88 (*p* = 0.810)	16.00 (*p* = 0.800)	−5.40 (*p* = 0.752)	−19.22 (*p* = 0.831)
Age × Meter-version			15.26 (*p* = 0.531)	63.09 (*p* = 0.622)
constant	3336.39 *** (*p* = 0.000)	10,888.49 *** (*p* = 0.001)	3614.53 *** (*p* = 0.000)	11,972.23 ** (*p* = 0.006)
N	99	99	99	99
$R^{2}$	0.306	0.136	0.3113	0.1415
*F*	13.97	5.00	8.41	3.07
*Prob* > *F*	0.000	0.003	0.000	0.013

Comments: *p*-values are reported in parentheses. *** designates statistically significant at the 0.001 level; ** designates statistically significant at the 0.01 level; and * designates statistically significant at the 0.05 level. Meter-version is a dummy variable that equals 0 in the miles treatment and 1 in the meters treatment. Female is a dummy variable that equals 0 for males and 1 for females.

## Data Availability

The raw data supporting the conclusions of this article will be made available by the authors on request.

## Appendix A. Analyzing Possible Order Effects

	**(1–Walking Scenario)**	**(2–Driving Scenario)**
Dependent variable	Distance Walking	Distance Driving
Independent variables		
Meter-version	−1908.02 *** (*p* = 0.000)	−7581.28 *** (*p* = 0.001)
Walking-first	583.09 (*p* = 0.177)	1306.13 (*p* = 0.559)
Walking-first X Meter-version	19.11 (*p* = 0.975)	2862.09 (*p* = 0.368)
Constant	2903.81 *** (*p* = 0.000)	10,495.7 *** (*p* = 0.000)
N	99	99
$R^{2}$	0.3142	0.1667
*F*	14.51	6.34
*Prob* > *F*	0.000	0.001
Comments: *p*-values are reported in parentheses. *** designates statistically significant at the 0.001 level. Meter-version is a dummy variable that equals 0 in the miles treatment and 1 in the meters treatment. Walking-first is a dummy variable that equals 0 if the driving scenario was presented first and 1 if the walking scenario was presented first. Walking-first X Meter-version is the interaction between these two dummy variables.		
